# Working Conditions of Occupational Physicians—A Scoping Review

**DOI:** 10.3390/ijerph19106222

**Published:** 2022-05-20

**Authors:** Eva Eisch, Paulina Kuper, Lara Lindert, Kyung-Eun (Anna) Choi

**Affiliations:** 1 German Economic Institute, Research Unit Vocational Participation and Inclusion, Konrad-Adenauer-Ufer 21, 50668 Cologne, Germany; eisch@iwkoeln.de; 2 Center for Health Services Research, Brandenburg Medical School Theodor Fontane, Fehrbelliner Str. 38, 16816 Neuruppin, Germany; paulina.kuper@mhb-fontane.de (P.K.); lara.lindert@mhb-fontane.de (L.L.); 3 Health Services Research, MIAAI Group, Faculty of Medicine/Dentistry, Danube Private University, Steiner Landstr. 124, 3500 Krems an der Donau, Austria

**Keywords:** occupational health, employee health, resources, stressors, prevention

## Abstract

Occupational physicians (OPs) offer a wide range of health support for employees and are confronted with varying job characteristics and demands. They monitor occupational health and safety and promote work(place)-related health measures and assessments. While helping employees to (re)gain a healthy status, their own job satisfaction as well as the investigation of their working conditions have earned limited research attention. Thus, this scoping review aims to summarize the current state of knowledge concerning OPs’ working conditions, i.e., work-related resources and stressors. PubMed, Web of Science and LIVIVO as well as grey literature were screened for relevant English or German articles until 10/2021. From a total of 1683 identified publications, we analyzed 24 full text articles that fulfilled all inclusion criteria. The overall study sample included 3486 male (54.6%), 2892 female (45.3%) and 5 diverse OPs, from which 1049 OPs worked in full-time (85.6%) and 177 in part-time (14.4%). The majority (72.4%) worked for the Occupational Health Service (OHS), 13% were self-employed, and 14.6% worked for a company/in-house service. The classification of stressors and resources was based on an inductively generated categorization scheme. We categorized 8 personal, relational and environmental resources and 10 stress factors. The main resources were support for personnel development and promotion, positive organizational policy, promoting work-life balance and other aspects of health. Key stressors were information deficits, organizational deficiency and uncertainty as well as socioeconomic influences and high professional obligations. The working conditions of OPs are still a topic with too little research attention. This scoping review reveals several starting points to maintain a healthy OP workforce and gives recommendations for action for the near future.

## 1. Introduction

Over the past 20 years, there has been an increasing demand for good solutions in occupational health [1]. Corporate and environmental structures have changed due to demographic developments: the workforce is aging and diversifying [2]. Along with the changing workplace conditions of employees (stronger shift towards workplace health promotion, demographic change and increase of mental illnesses), new challenges for OPs of the 21st century arise. A major challenge for occupational health services is the need for the development of structures and ways of working that maintain (1) integrity in a more commercialized environment and (2) both the quality of service and the attractiveness of the profession in the long term [3]. Higher general health status of employees is a major asset for companies to yield better productivity and competitiveness. “Occupational safety and health includes all measures which ensure and improve work safety and health protection. Operational management of occupational safety and health is responsible for identifying needs, making decisions and eventually taking measures” [4]. In general, occupational physicians (OPs) may play a crucial role for the prevention and rehabilitation of employees. Through workplace inspections and consultations with employees, most OPs have a thorough knowledge of working conditions and often a direct access to change specific work demands. However, this constellation often creates a perceived tension in the triangle employee-employer-physician with un-communicated reservations regarding the representation of interests. 

Even though OPs in Germany make up a rather small proportion of all working physicians in Germany [5], their far-reaching involvement on a personal, organizational, but also political level is nevertheless evident. In Germany, the general conditions of OP service are ruled in the “Act on Occupational Physicians, Safety Engineers and Other Occupational Safety Specialists” [6]. The type of employment of an OP depends fundamentally on the respective circumstances of the company [7]. In principle, there are several employment relationships: (full or part-time) salaried OPs, freelance OPs and physicians that work for an intercompany occupational health service. A full-time OP is usually found in large companies or in companies with high-risk potentials (e.g., in a chemical plant or in the Armed Forces) to find specific measures for their requirements. Outsourced solutions use freelance OPs, often in small and medium-sized enterprises (SMEs). Working for the inter-company occupational health service, an OP may also join a registered association or an independent service company that provides the medical care.

Despite their important role as investigators of work-related risk factors and as a creator of healthy working conditions, OPs are also active employees themselves, thus, equally exposed to potential work-related risk and occupational hazards. The research indicated that agency and well-being, as well as health status, play a crucial role in the experienced quality of and satisfaction with work. Health workers are often exposed to special stresses and complain about a high burden of inadequate work conditions. They are particularly frequently affected by absenteeism due to mental disorders [8]. Their mental health and wellbeing should be of special interest not only regarding their personal health, but also as their mental health and wellbeing affects the quality of care [9,10]. Especially during the global pandemic caused by the coronavirus disease, healthcare workers were confronted with great psychological distress [11,12,13,14].

The World Health Organization (WHO) defines health as “ a state of complete physical, mental and social wellbeing and not merely the absence of disease or infirmity” [15]. Job demands that can affect employees’ mental health are, for example, leadership, organizational justice, effort-reward imbalance, atypical forms of employment, social support or job insecurity [16]. In healthcare workers, job satisfaction and subjective wellbeing is associated with workplace performance, retention, instability of available workforce and shortage of healthcare workers [17]. Moreover, heavy workloads, long shifts, a high pace, lack of physical or psychosocial safety, chronicity of care, moral conflicts, job (in)security, social support and bullying can result in psychological distress, leading to burnout, depression, anxiety disorders or sleeping disorders [10,18,19,20,21]. 

The idea of working conditions impacting employees’ health is not new. Two still widely used models that provide a theoretical background, are the transactional stress model and the job demand control (JDC) model. Using job satisfaction and the concept of well-being are more recent approaches in investigating work conditions. 

### 1.1. Transactional Stress Model 

To explore the OPs’ working conditions, we use the indicators “stressors” and “resources”. According to the transactional stress model of Lazarus and Launier [22] (1981), stress mainly depends on the cognitive evaluation and coping strategies that the individual may apply to process environmental stimuli. According to their model, stressors are not only reduced to external stimuli, but take also individual intrinsic factors into account. The three essential stress-relevant relationships are: harm/loss, threat and challenge. The choice of the coping strategies belongs to the most important aspect in the individual handling of stress and has a significantly greater influence on health or illness than the actual stress episodes. There are two main categories to cope with the stressors: (a) change of the stressed transaction (instrumental) and (b) the regulation of emotion (palliation). The instrumental coping has a direct influence on the stressful situation. Under the use of acquired skills and information, the problem is solved or the obstacle removed. Palliative coping, on the other hand, aims at regulative emotions such as relaxation. In the best case, negative emotions such as fear or anger should be controlled and diminished.

The selection of coping strategies described above depends on a variety of circumstances: situational context or the environment determines which form of coping is appropriate and at the same time most promising. The degree of (perceived) helplessness can also be a factor influencing the choice of coping strategies. Furthermore, Bamberg et al. [23] (2003) have demonstrated with their model that the development of stress is not merely attributable to the individual person- and condition-related factors, but is rather based on their mutual interaction. 

### 1.2. Job Demand Control (JDC) Model

Another widely used model is the Job Demand Control (JDC) model by Karasek and Theorell [24] (1990). He conceptualized mental stress as a result from the amount and intensity of job demands and the amount (time and intensity) of work, as well as from the freedom of action and decision (latitude) of the respective employee. Karasek and Theorell understand the combination of work requirements and decision latitude as a two-dimensional model from which four different types of psychosocial perception of an occupation can result: high-strain jobs, active jobs, low-strain jobs and passive jobs. The four dimensions of the model vary between different types of psychosocial stress depending on the extent of potential work intensity and the potential scope for action. In addition, a third dimension was added to the JDC model at the end of the 1980s: the influence of social support. Karasek and Theorell predict a combination of low social support, low decision-making latitude and high decision-making potential, a low degree of decision-making freedom and, at the same time, high work demands and a high stress level for employees [24]. One of the most important conclusions of the JDC model, according to Karasek, is the possibility to improve the work-related mental health of employees without affecting the productivity of the organization: It appears that the stress of the workplace can be reduced by increasing decision-making latitude, irrespective or independent of changes in job demands. The key point lies, accordingly, in the employee’s ability to make important decisions about his or her work.

### 1.3. Job Satisfaction 

A significant model in understanding work conditions is the concept of job satisfaction, which has been linked to both organizational behavior and physical and mental health [25]. Due to the shortage of nurses, this concept has been widely used to investigate how the nursing workforce can be stabilized. The main attributes job satisfaction gained via concept analyses show that “fulfillment of desired needs within the work setting”, “happiness or gratifying emotional responses towards working conditions” and “job value or equity” are key features impacting the overall job satisfaction. These attributes interplay with demographic, structural work characteristics and environmental variables [25]. 

### 1.4. Concept of Psychological Well-Being

Another widely used and early concept to understand work condition is the concept of psychological well-being [26,27]. Previous work has investigated its influence on vocational identity and career commitments [28,29]. Higher socioeconomic status contributes to better health and well-being and different types of work may also predict the perceived level of well-being, with work and educational experiences being the strongest predictors of well-beings, especially among older adults [30]. Linking psychological well-being to physical health, biological regulation and neuroscience delivers additional objective indicators for more general benefits [31]. Tools to assess psychological well-being have now been incorporated into many fields in order to describe challenges and transition periods such as adolescence or new job opportunities.

Staying healthy at work is highly relevant. OPs are assigned with a large number of specific tasks (e.g., including numerous legal foundations/basis, diverse employment relationships, working hours). The aim of the current scoping review is to assess the current knowledge concerning OPs’ working conditions. Which specific work-related resources and stressors have been reported? The presented resource and stress models are applied to analyze the extracted working conditions. 

## 2. Materials and Methods

This scoping review was conducted by a multidisciplinary team with proven experience in health services research, psychology and rehabilitation science. The review process comprised (a) the identification of the research question, (b) identification of relevant studies, (c) study selection, (d) data extraction (mapping) and (e) data syntheses and reporting of results. We herein followed established reporting guidelines for scoping reviews (PRISMA P; PRISMA ScR) [32,33]. 

### 2.1. Identification of Research Question

We address the following research questions:

What are the reported working conditions for OPs, described in international publications over the last 20 years?

To what extent are work conditions described as stressors or resources in this context?

### 2.2. Identifying Relevant Studies, Inclusion and Exclusion Criteria

To identify relevant studies, we will follow the Population-Concept-Context (PCC) framework (see Table 1), recommend by the Joanna Briggs Institute [34] (JBI Manual for Evidence Synthesis, JBI, 2020). 

This review includes all types of experimental studies, observational/quasi-experimental studies, cross-sectional studies, case studies, and all types of (systematic) reviews published in German or English until the end of 10/2021. There were no restrictions concerning country or region of the world. Additionally, grey literature was hand-searched. Opinion papers, editorials and commentaries were excluded. 

Since the initial search showed only a limited number of references, we decided to widen the inclusion criteria as much as possible with respect to the PCC criteria. Articles that were excluded from this study mainly focused on employee health and health professions other than OPs (e.g., general practitioners or nurses) only. Findings that provided separate information on OPs’ work conditions were still included. All articles meeting the defined criteria of the PCC framework (see Table 1) were included.

### 2.3. Study Selection 

Included databases were Medline (PubMed), Web of Science and LIVIVO. The search strategy contained keywords and subject headings from the PCC framework. According to the JBI approach, the search strategy followed a three-step selection. An initial, limited search in set databases was conducted with following predefined keywords and index terms. The search algorithms were:

#1Population: (Betriebsärzt* OR Betriebsarzt OR Arbeitsmediziner* OR “company doctor” OR “company physician*” OR “industrial physician*” OR “company medical officer*” OR Werksarzt OR Werksärzt* OR “work* doctor*” OR “occupational health physician*” OR “occupational physician*”)

#2Outcome: (Belastungsfaktor* OR Belastung* OR Arbeitsanforderung* OR Arbeitsintensität OR Handlungsspielraum OR Tätigkeitsspielraum OR “decision latitude” OR “job demand*” OR “work demand*” OR demand* OR burden OR resource* OR “soziale Unterstützung” OR “social support” OR Beanspruchung* OR stressor* OR Stressfaktor* OR “load factor*” OR “stress factor*” OR “job related resource*” OR “work related resource*” OR workload OR “job control” OR “job stress” OR “work stress” OR Arbeitsbedingung* OR “working condition*” OR Arbeitssituation OR “work situation”)

**Figure 1:**
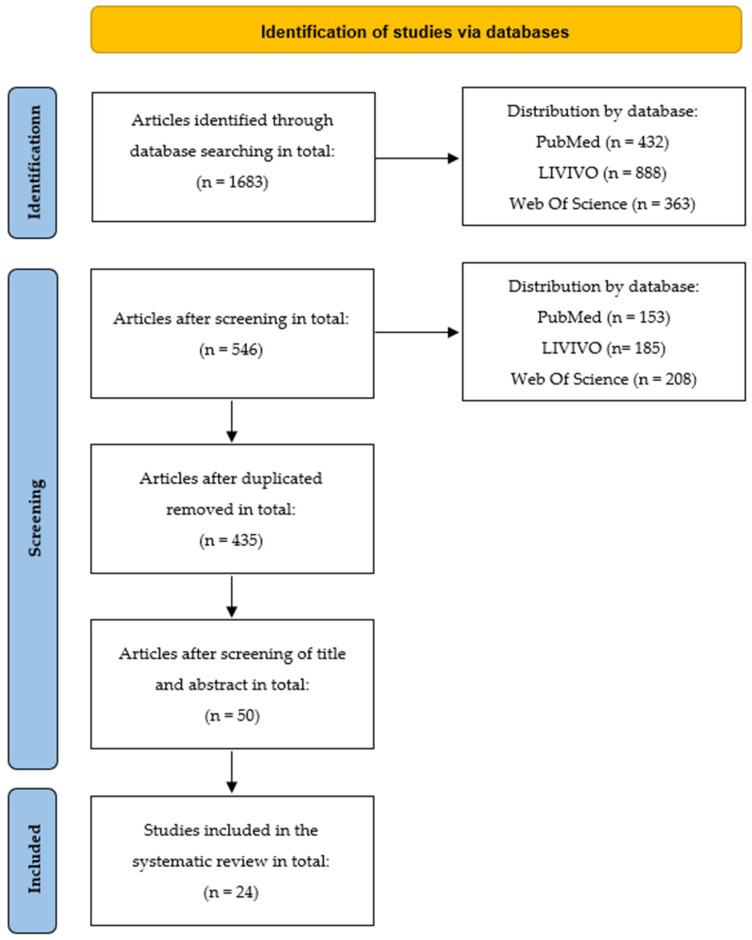
PRISMA 2020 flow diagram according to Page et al. [35] (2020). Flow diagram of the scoping review, which included searches of databases only.

Consecutively, both algorithms were combined by the Boolean operator “AND”. Retrieved articles were screened for additional keywords and index terms. A second search including all identified keywords and index terms followed, but yielded no additional results. Following a snowball principle, reference lists of included studies were screened for additional sources. Retrieved articles were imported to Endnote X9 and exported to Rayyan (Cambridge, USA) for the title and abstract screening. First, three reviewers (E.E., L.L. and K.-E.C.) independently conducted a title and abstract screening. Disagreements were reflected and discussed in the team and solved by consensus. The full text screening followed the principles of title and abstract screening. Figure 1 illustrates the full study selection process.

### 2.4. Data Extraction (Mapping)

We used a data extraction chart in Microsoft Excel tailored to the objectives of this review (see Table 2). The chart was piloted by E.E. and adjusted by E.E., L.L. and K.-E.C. in an iterative process. Relevant data of each article were independently extracted by at least two researchers (E.E., L.L. and K.-E.C.). Results were discussed and harmonized in the overall team (E.E., L.L., K.-E.C. and P.K.) led by P.K.

### 2.5. Data Synthesis and Reporting 

For data synthesis and reporting we used an inductively generated categorization scheme for resource and stress factors that was piloted by E.E. and adjusted by all study team members in an iterative process. E.E. synthesized and reported all relevant data (top category, description/definition, attribution and total N—assigned studies in absolute frequency) of each article. Subsequently, all results were cross-checked with P.K. for comprehensibility and consistency.

## 3. Results

The surveyed studies included a total of 3486 male (54.6%), 2892 female (45.3%) and 5 diverse OPs, from which 1049 OPs worked in full-time (85.6%) and 177 in part-time (14.4%). The majority (72.4%) worked for the OHS, 13% were self-employed and 14.6% worked for a company/in-house service. The average age was high (1.1% younger than 36 years, 4.7% being between 36 and 45 years old, 78.9% aged 45 to 55 years and 15.6% aged over 55 years).

Table 3 summarizes the most important extraction results of the review. Further information and the entire table of extraction results can be found in the appendix (Table A1 and Table A2). The presentation of results follows the alphabetical order of the first author’s last name. With the exception of some Japanese, Turkish and international studies, most of the found evidence were reported by European groups.

### 3.1. Identified Resources

Table 4 displays identified resources of the OPs. We categorized eight resources, some of which also loaded negatively as stressors: social interaction, perceived repuation of the profession, characteristics of the emploment relationship and scope for decision-making/action (see Table 5). Other factors were only identified to be loaded positively, including aspects of health, work-life balance, opportunities for personnel development and promotion, as well as organizational policy. 

### 3.2. Identified Stressors

Table 5 displays identified stress factors of the OPs. Socioeconomic factors, perspectives, information deficits, organizational complication, uncertainty factors and professional obligations were identified as stressors that had no positive loadings. 

## 4. Discussion

Given the declining number of employed OPs, there is a need to focus on eliminating stress factors and emphasizing resources in order to increase the overall attractiveness of the occupation. We identified 8 resources and 10 stressors (personal, relational and environmental factors) for OPs. Of those, some factors loaded both positively as well as negatively (i.e., social interaction, reputation of the profession, characteristics of the employment relationship and scope for decision-making/action). Support for personnel development and promotion, positive organizational policy, promoting work-life balance and other aspects of health were the main resources. Information deficits, organizational deficiency and uncertainty were key stressors besides socioeconomic influences and high professional obligations. 

The majority of the surveyed OPs worked for the OHS (72.4%). Earlier studies have found that the type of employment may cause very different working conditions for OPs (e.g., [39,58]). A direct comparison of the employment relationships shows that self-employment and/or part-time work represents a resource for OPs, whereas employment with the occupational health service and/or full-time work can be classified as a stress factor. One reason for this could be that full-time employment in the company is only an option for an OP, when the number of hours worked exceeds 1640 per year, and the hurdles for a freelance existence are too great. Freelance work is often associated with less social interaction and peer feedback. Of course, the employment relationships and the functions of the individual OPs may vary in their characteristics from country to country.

The majority of the OPs was over 45 years old (95.5%), with one in seven being over 55 years. This fits with the observation that the next generation of OPs is lacking in occupational medicine. Accordingly, the stress factors of occupational medical practice should be critically scrutinized and remedied as best as possible. Above all, the organizational difficulties, and the prejudiced assessment of the occupational profile by external parties can be counteracted with educational work.

An essential influence for the appraisal of stress or resource was the amount of decision-latitude and agency. This is because it is evident, also according to the JDC model, that the highest levels of satisfaction are found in active jobs where there are high job demands but also opportunities for the use of authoritarian actions and decisions. The Federal Institute for Occupational Safety and Health (German: Bundesanstalt für Arbeitsschutz und Arbeitsmedizin, BAuA) sees great potential for action in education and training. In particular, the steady shift from pure occupational health and safety measures toward workplace health promotion is leading to numerous new fields of application that place the occupational health professional in the foreground as a central actor [59]. Compared with other specialties, there are also many structural advantages for OPs. For example, most OPs do not have to work nights, shifts or on weekends. Their average hours worked per week are lower than those of hospital doctors [37], who work up to 59 h per week, which may in turn endanger their own health. More attention should be paid to the field of occupational medicine already in medical school: Continuing education should be subsidized and the advantages of the profession over other, clinical health professions should be discussed.

The working conditions of OPs are still a topic with too little research attention. One possible reason may be that OPs account for only 3.3 percent of all working physicians in Germany (in comparison, hospital physicians, for example, account for more than 50 percent) and thus do not justify a sufficient need for research [60]. Since OPs take a fundamental role in the diagnostics and management of employee health, a closer look at work-related stress factors as well as the resources of OPs is essential, as both occupational health systems as well as companies and their employees can benefit from healthy and satisfied OPs [47]. 

Psychological well-being as well as job satisfaction are widely used to identify work conditions as work-related stressors and resources [16]. Therefore, both may be key concepts to elucidate work-related stressors and resources for OPs. Since OPs work in multidisciplinary teams and are perceived as situated in a triangle between patient–employer–physician, further research would be necessary to illuminate the resulting specific demands and resources. Only if we ensure that the working conditions for OPs are appropriate, we can assume that they are also able to contribute to an adequate occupational health service, not at least in the sense of organizational health literacy. 

### Strengths and Limitations

To our knowledge, this scoping review is the first systematic and standardized overview of the working conditions of OPs. Our recommendations may be perceived to be not yet specific enough. Evidence-based recommendations for practice need a sufficient level of scientific knowledge. Considering our broad approach, the total amount of findings was very limited. The above-mentioned aspects demonstrate that there is a clear need for research (including prospective trials), which must be implemented in the future. 

One possible limitation of our scoping review is that it mainly covers European and some Asian studies. Occupational health solutions vary across countries, of course. It would have been interesting to include more (national) grey literature such as guidelines, website information of associations and company in-house information. Moreover, language restriction may have biased our results. 

## 5. Conclusions

The nature and characteristics of OPs’ work significantly differs from that of other medical professions due to numerous framework conditions. This scoping review delivers concrete indications for science and practice to counteract potential stress factors and strengthen resources perceived by OPs. Regarding the targeted survey of the working conditions of OPs, there is a need for a larger number of more objective procedures that are not exclusively based on a questionnaire-based, subjective self-assessment.

Only with an occupational health promotion with targeted support of Ops can the next generation of OPs can be secured. “Individual physicians will benefit, the organizations employing those physicians will benefit, and so too will the occupational health systems and the workers” [47].

Inspired by on our findings, we would like to propose the following recommendations for action for the near future:

1. Drive research forward (both people- and practice-oriented);
2. Secure the next generation (greater expansion and emphasis on occupational medicine in medical studies);
3. Eliminate information deficits (promote continuing education in occupational medicine);
4. Optimize interdisciplinary teamwork (e.g., with occupational safety specialists or family physicians);
5. Eliminate prejudices (education/public relations work);
6. Make capital available (discourse on a national, political level);
7. Emphasize the resources of OP activities give greater priority to the issue of prevention in the company.

## Figures and Tables

**Table 1:** PCC characteristics.

Criteria	Characteristics
Population	- Occupational/Company physicians - All employee status (part-time, full time, inside and outside the company)
Concept	- All work conditions (personal, social) - All stressors, resources related to work
Context	- All sectors - All countries - Publication year: 2009–2021 - Publication available in English and/or German

**Table 2:** Chart elements and associated questions leading the extractions.

Chart Elements	Associated Questions
**Publication Details**	
Author(s)	Who wrote/published the article?
Year of publication	When was the article published?
Country of origin	Where was the study conducted and published?
Publication type	What type of publication is this?
**Study details**	
Aim(s) of the study	What was/were the aim(s) of the reported study?
Methodology	What design/methods were used?
Sample	Who was the target population (sociodemographics) and how many were included in the study?
Work conditions	Which work conditions were of primary interest?
Results Level of evidence	Which results were observed/obtained? What is the level of evidence according to COCHRANE for this study?
**Conceptualization**	
Measure	What measures were taken? Which outcomes were obtained?
Identified loading factors and stress factors	Which stress/loading factors were identified?
Identified resources	Which resources were identified?
Strengths and weaknesses of the study	Which potential biases and strengths do we detect? Were there any reported limitations or quality issues?

**Table 3:** List of included studies and most relevant parts of data extraction.

Reference, Country of Origin	Sample and Research Design	Outcome (Objectives/Aim)	Results
Alaguney et al. [36] (2020) Turkey	n = 478 physicians of which: n = 251 with demonstrated work experience as an OP (response rate: 10.34%) Cross-sectional study	Underreporting of occupational diseases (online questionnaire: 30 questions and statements with 5-point Likert scales from 1 = not important to 5 = very important)	(a) Fear of potential job loss as a result of occupational disease screening and diagnosis; for themselves (*p* = 0.015); for workers diagnosed with an occupational disease (*p* = 0.015); (b) inadequate examination of occupational safety and health and limitation of detection opportunities of occupational diseases in the workplace environment (*p* = <0.001); (c) insufficient awareness among physicians of work-related diseases and their under-reporting (*p* = 0.043)
Cakir and Ilhan [37] (2018) Turkey	n = 258 active working OPs in Ankara (response rate: 100%) Cross-sectional study	Working conditions (questionnaire: 85 questions) of which: Intrinsic, extrinsic and general job satisfaction (20 questions: Minnesota job satisfaction scale (MJSS) consisting of 5-point Likert scales ranging from 1 = very dissatisfied to 5 = very satisfied)	(a) Average monthly working time of an OP: 143 h (36 h/week); (b) 80.6% of OPs consider their work to be suitable and meaningful; (c) 27.1% rate their occupational physician education/training as satisfactory or sufficient; (d) 86.3% of the respondents perceive the subordination to the employer as unpleasant; conflict of interest between payment and self-determined action; restriction of freedom of decision; (e) Significant positive relationship between extrinsic satisfaction and monthly salary (*p* < 0.001); (f) significant positive relationship between number of employees to be under an OP’s care and overall satisfaction (*p* = 0.013; (g) significant positive relationship between years worked as OP and intrinsic satisfaction (*p* = 0.009)
Demou et al. [38] (2018) International	Rating-round 1: n = 332 OPs Ranking-round 2: n = 232 OPs (both with unknown response rate) Delphi survey (second rounds over all) First round: rating Second round: ranking (questionnaire-based) First and second round: same pool of persons, but irrespective of participation in round 1	Job requirements and practice competencies by activity area; systematically developed questionnaires Rating: ranking the importance of 12 competency areas for the individual activity (1 = not important to 5 = very important) Ranking: generation of a ranking of the previously listed areas with the addition of newly proposed items from round 1)	First round (rating): (a) most important competence for all groups: good clinical care (M = 4.56, SD = 0.20); (b) least important competence for all groups: teaching and educational supervision (M = 3.81, SD = 0.09) Second round (ranking): (a) top 3 rankings of all groups: (1) good clinical care; (2) general principles of assessment and management of occupational hazards to health; (3) assessment of disability and fitness for work; (b) lowest rankings of all groups: (1) management competences; (2) competencies on teaching/educational supervision; (c) for all other areas, there are clear differences according to the individual areas of occupational activity; (d) consent: all 12 domains were regarded as important (90% and over)
Glaser et al. [39] (2015) Austria main study	n = 147 OPs (unknown response rate) Cross-sectional study (Online-questionnaire)	Everyday work; work tasks; general loads/stress factors; cooperation with other occupational groups; professional identity; (online questionnaire with 221 items consisting of five-point Likert scales from 1 = no, not at all to 5 = yes, exactly)	Everyday work/work tasks/general loads/stress factors: (a) dissatisfied with the range of training/education opportunities (n = 128); (b) lack of young professionals (M > 3); low status/relevance of occupational medicine in medical study/training (M = 4.2); (c) resistance to change on the part of employers (M > 3.5); (d) difficulty in measuring the success of implemented interventions (M > 3.5); (e) high documentation effort (M > 3); (f) competitive situation in occupational health services higher (M > 3) than for self-employed (M < 3); competition overall (just) little stressful; (g) different burdens according to employment relationship Cooperation with other occupational groups: (a) classification/rating of cooperation as “meaningful” (M > 4); (b) competition with safety professionals and occupational psychologists is low (M < 2) Professional identity: (a) rather dissatisfied with appreciation on the part of society (M < 3); (b) relatively satisfied with appreciation from employees (M > 3)
Glaser et al. [39] (2015) Austria pre-study	n = 6 OPs (unknown response rate) Qualitative cross-sectional study	Everyday work; work tasks; general loads/stress factors; cooperation with other occupational groups; professional identity (deductive expert interviews; qualitative content analysis)	Everyday work/work tasks/ general loads/stress factors (mentions): (a) insufficient information which is necessary for work (n = 3); (b) role conflict between reporting and confidentiality (n = 2); (c) payment in relation to workload is adequate (n = 4); (d) insufficient acceptance by employers and employees (n = 2); (e) initiation, implementation and evaluation of changes in the company is problematic (n = 2); (f) lack of junior staff (n = 4); (g) other named loads/stresses: physical stress, weather aspects, no clearly defined role as OP and difficulties in communicating/teaching about prevention in relation to the workplace Cooperation with other professional groups (mentions): (a) positive evaluation of cooperation (n = 3); (b) cooperation among OPs rather negative (n = 3) professional identity (mentions): (a) competencies to bring along: impartiality, expertise and openness (n = 5); (b) utopian expectations on the part of employers (n = 5); (c) professional appreciation (n = 2); (d) social recognition (n = 4)
Gross et al. [40] (2012) England	n = 145/224 NHS (national health service) OPs (England, Scotland, Wales) with responsibilities for health care workers; ANHOPS (Association of National Health Occupational Physicians) membership (response rate: 65%) Cross-sectional study (postal questionnaire-based)	Determine the experience and training in identifying substance misuse among health care workers	(a) Only a small proportion of OHPs felt adequately trained in the assessment (39%), detection (37%) or treatment (12%) of substance misuse and few used standardized addiction screening tools or brief interventions in routine practice; (b) OPs were unfamiliar with dedicated services for addicted health care professionals and with local specialist NHS addiction services, and felt resources and support available to them were limited
Gyo et al. [41] (2016) Germany	n = 136 (1992) to n = 86 (2012) OPs in Germany (unknown response rate) descriptive correlation study	Number of state-certified OPs in relation to socio-economic data in Germany (manual research on various internet platforms or databases)	(a) Sharp decline in the number of OPs in Germany from 1992 to 2012 (decline rate: 37%); (b) positive correlation between the decrease in OPs and the increase in GDP in Germany (r = 0.47); government expenses concentrate on other areas; (c) uneven distribution of employed OPs in the discrete federal states (2012): Saarland: 8 OPs per 1 million employees; North Rhine-Westphalia: 0.8 OPs per 1 million employees
Hobson et al. [42] (2016) England	n = 2 OPs working in the private sector Occupational Health Service (OHS). A total of 108 accompanied consultations matched to 103 non-accompanied consultations (unknown response rate) Prospective, unblinded and observational (over the course of 16 months)	A total of 108 accompanied consultations matched to 103 non-accompanied consultations; ill health retirement; diagnosis; complexity; time (duration); consultation process indicators; the consultations occurred in clinics held in a number of different locations and included referrals from the public and private sectors and from a variety of workplaces. Public sector referrals were predominantly from two large local authorities	(a) Accompanied consultations more likely to be connected with: ill health retirement (*p* < 0.01); neurological diagnosis or multiple diagnosis (*p* < 0.01); rated as complex (*p* < 0.01); taking longer than 30 min (*p* < 0.01); (b) 54% of companions were spouse/partner (of patient); (c) an impact by the companion was recorded in 81% of consultations; (d) in 36% of consultations the impact of the companion was helpful or in agreement with the advice provided by the OPs; (e) in 28% of accompanied consultations interruptions were recorded; (f) 6% of consultation: consultation or companion was difficult; (g) 10 accompanied consultations where companion was a trade union representative was male: 80% (*p* < 0.05); but only 12% of consultations were rated as complex; half of the consultations: trade union representative provides information; in 30% of consultations: interruptions and asked questions
Hoedeman et al. [43] (2010) The Netherlands	n = 43 OPs (response rate: 97.2%) and n = 489 sick workers with mild and severe medically unexplained physical symptoms (MUPS) (unknown response rate) Cross-sectional study	Consultation load; difficulties and needs of OPs in the course of sickness certificate of employees with severe MUPS (Utrecht burnout scale and Utrecht work engagement scale)	(a) OPs do not need more time for workers with severe MUPS than for workers with low MUPS (*p* = 0.266); (b) communication difficulties with the treating primary care physician for workers with severe MUPS (OR = 5.42, *p* < 0.01); (c) relevant confounding factors: attribution of physical symptoms to somatoform causes (*p* = 0.005) and to patient age (*p* = 0.029)
Hoedeman et al. [44] (2010) The Netherlands	n = 6 RCTs (randomized controlled trials) with a total of 449 patients; RCTs concerning consultation letters for patients with MUPS (medically unexplained physical symptoms) (unknown response rate)Qualitative study (intervention/systematic review)	A total of 2 authors screened the abstracts of the studies + independently assessed the risk of bias of the included studies, objectives to assess the effectiveness of consultation letters to assist primary care physicians or OPs in the treatment of patients with MUPS and diagnostic subgroups	Final conclusion: CL may be helpful for physicians who treat patients with MUPS (based on the provider-related outcomes)
Koike et al. [45] (2019) Japan	OPs working in full-time: 2002–2004: n = 578 to 2012–2014: n = 953 (unknown response rate) Cohort study/ longitudinal study	Retention rate/trends of OPs and factors associated with it (semiannual survey dates through censuses of physicians from 2002 to 2014)	(a) Retention rate from 2012 to 2014: 76% (24% of OPs stopped working full-time); (b) the chance to continue working as a OP decreases when working in a small town or village (*p* < 0.05); (c) the chance to continue working as a OP decreases if the OP has been working for more than 41 years (*p* < 0.05); (d) the chance to continue working as a OP increases if a OP has already been registered as a OP in > 2 consecutive survey periods (*p* < 0.001)
Lalloo et al. [46] (2020) Scotland	n = 213/1207 practicing UK Ops (response rate: 18%) Online survey	Current and former research-activity; current and former teaching activity; demographics; qualifications; career profile; research-related attitudes; dissertation experience	(a) 162 (76%) undertook research at some career-point, of which 44 (27%) were currently research-active; (b) 154 (72%) undertook teaching at some career-point, of which 99 (64%) were currently teaching active; (c) of those who had never undertaken research (n = 51) or teaching (n = 59), 40% and 42% were interested in doing so; (d) key barriers: lack of time and opportunity; research activity was higher in healthcare OPs compared to industry OPs
Lesage et al. [47] (2013) France	n = 1670/5010 OPs working in France (by French ministry of labor) (response rate: 33%) nationwide cross-sectional study (online questionnaire)	Maslach burnout inventory (emotional exhaustion, depersonalization, feelings of low personal accomplishment); perceived stress scale (stress level); primary appraisal of identity scale (identity threat; job characteristics	(a) 11.8% burnout compared to 5% in French general practitioners (main characteristic of the burnout pattern: feelings of very low personal accomplishment: 63.9%); (b) weak correlations with job characteristics; (c) stress and identity threat correlating with all three dimensions of burnout; (d) perceived stress-> main risk factor for emotional exhaustion and identity threat for feelings of low personal accomplishment
Ljungquist et al. [48] (2015) Sweden	n = 481 OPs; n = 4257 GPs (general practitioners); n = 9452 physicians working in other clinical settings; overall: n = 22,349 physicians (not all included in the 3 groups above) (response rate: 60.6%); Questions mailed to all of the 36,898 physicians working and living in Sweden (October 2008); all physicians who stated they had consultations concerning sickness certification at least once a month constituted the study group (n = 14,190) Cross-sectional study (paper-based)	Work situation of OPs regarding handling of sickness Certification compared with other physicians, in particular general practitioners (GPs); associations between OPs’ experiences of assessing and providing a long-term prognosis of patients’ work capacity and some potentially interrelated factors; 163 questions about physician’s work with sickness certification mailed to home address; 11 items on sickness certification and general work	(a) 46% of OPs had a well-established workplace policy and substantial support from their immediate manager regarding sickness certification tasks, compared with GPs (32%) and especially with physicians working in other clinical settings (14%); (b) collaborations with other team members, with the Social Insurance Agency, and, most of all, with employers, was much more frequent among OPs than among GPs and among the other physicians (employers: 76%); (c) 43% of OPs finding it problematic to handle sickness certification at least once a week (GP: 54%); (d) participation in coordination meetings concerning specific patients on a weekly basis was negatively associated with finding it ‘not at all/somewhat problematic’ to provide a long-term prognosis about patients’ work capacity; (e) OPs seem to have a more favorable work situation in their work with sickness certification; (f) experience of sickness certification consultations as problematic once a month or less often, not experiencing sickness certification tasks as a work environment problem, holding a specialty in occupational medicine, and having a well-established workplace policy regarding sickness certification matters were significantly positively associated with finding assessment of work capacity as ‘not at all/somewhat problematic’; (g) participation at least once a week in coordination meetings with the Social Insurance Agency and/or employer regarding sickness certified patients was negatively associated with finding assessing patients’ work capacity as ‘not at all/somewhat problematic’
Moriguchi et al. [49] (2013) Japan	n = 557 OPs; Kyoto occupational health promotion center; (response rate: 31% (175 OPs); n = 76 no longer active as OPs; n = 86 OPs who were either; private clinic-based or hospital-based questionnaires via mail in 2008	Examine activities of private clinical- or hospital-based OPs; identify difficulties encountered in occupational health service	(a) OPs wished to allocate more time for: examination follow-up (2.6 h/month); mental health care (2.0 h/month); prevention of overwork (1.9 h/month); attendance at the safety and health committee meetings in the plant (1.9 h/month); (b) discrepancy between the current and the desired allocation was greatest for: risk assessment (171% as the desired/current ratio); maintenance and management of work and the work environment (150 and 152%); time allocation for health examinations appeared to be sufficient; (c) major difficulties in: management of mental ill health (36 OPs); guidance of workers on sick leaves (11 OPs); followed by prevention of health hazard due to overwork (30 OPs); diagnosis of return to work (15 OPs); (d) OPs had difficulty in dealing with: industrial hygiene-related issues such as risk assessment (14 OPs) and maintenance and management of work and work environment (11 cases each; (e) respondents were generally self-confident regarding: physical health management (typically providing general health examinations); to solve the problems related to lack of experience with mental health issues referral to experts
Moriguchi et al. [50] (2020) Japan	n = 181 OPs; 2016: n = 946 OPs/postal addresses; overall: n = 363 responses; (response rate: 38%); of these, n = 139 no longer active as OPs; other cases excluded; usable answers: n = 181 OPs: (50% of the 363 respondents or 19% of the original 946 mail addresses were usable); work setting: private clinic-based: 131 OPs; hospital-based: 50 OPs questionnaires via mail in 2016 (and similar survey in 2008)	To compare the activities and encountered difficulties of Japanese part-time OPs in 2008 and 2016 and to investigate the effects of the stress-check program	(a) 62% OPs frequently encountered difficulties in stress-check-related activities in 2016; (b) many OPs reported difficulties in the mental health care and the prevention of health hazard due to overwork both in 2008 and 2016; (c) enforcement of the stress-check program in 2015 changed the activities of part-time OPs in Japan; (d) OPs wished to allocate more time for: prevention of overwork (2.3 h/month); General health examination (2.1 h/month); stress-check (2.1 h/month); Follow-up of examination (1.9 h/month); round of the work area (1.8 h/month); interviews with high-stress employees (1.2 h/month); management of stress-check system (1.4 h/month); (e) discrepancy between the current and the desired allocation greatest for: development of comfortable workplaces; health promotion activity and health and hygiene education; time allocation for fields related to periodical general health examinations appeared to be sufficient; (f) in 2016, difficulties were experienced most often in: stress-check (112 OPs); followed by mental health care (66 OPs); prevention of health hazard due to overwork (61 OPs); diagnosis of return to work (38 OPs); OPs encountered difficulties more in interview with high-stress employees than management of stress-check system; (g) proposals were made by 39 OPs: increase of training course for information exchange of experiences with experts (9 OPs); sharing roles of mental health issues with psychiatrists (8 OPs)
Moßhammer et al. [51] (2012) Germany	n = 23 primary care physicians/OPs (unknown response rate) Qualitative cross-sectional study	Cooperation/communication among primary care physicians and OPs in Germany: deficits and barriers (focus group interviews using semi-standardized interview guidelines and qualitative content analysis)	(a) Existing deficits on the topics: work disability, chronic diseases and reintegration of workers; (b) mentioned/named barriers: fear, mistrust, prejudices, lack of legal regulations and lack of knowledge regarding the respective other occupational group; (c) view of OPs on prejudices and ways of dealing
Nübling et al. [52] (2007) Germany	n = 356 OPs (unknown response rate) Cross-sectional study with the addition of comparative data from different occupational groups	(1) perception of workplace; (2) consequences of stress; (3) psychosocial workplace factors (Copenhagen psychosocial questionnaire (COPSOQ) with comparative data from different occupational groups)	(a) OPs perceive a significantly lower conflict between work and private life than many of the comparative occupational groups (scale mean = 42); (b) the scale of social relationships at work is rated below average by OPs (scale mean = 36); (c) job insecurity is very low among the group of OPs (scale mean = 23); (d) OPs feel significantly less affected by burnout in comparison with other occupational groups (scale mean = 37)
Nübling et al. [53] (2014) Germany	n = 777 OPs (response rate: 34%) cross-sectional study adding the reference groups “physicians in hospitals” and “average of all professions in Germany”	(1) work requirements/work situation; (2) health behavior; (3) support wishes for health prevention; (4) stresses and loads (COPSOQ + further items on health and job-specific stresses)	(a) Work-life balance represented a significantly more positive influence among OPs than among hospital physicians; (b) OPs have on average lower quantitative and emotional demands than hospital physicians; (c) quantitative requirements vary depending on the employment relationship; (d) competitive pressure very low; (e) scope for decision-making and development opportunities are very high among the OPs compared to the average of the comparison groups; (f) the level of job insecurity among OPs is very low; (g) low level of social relationships during work; (h) lack of social recognition; (i) the overall health behavior of OPs is better than that of the general population; (j) lower risk of developing burnout
Persechino et al. [54] (2016) Italy	Random sample of 1237 OPs, enrolled in the national register of OPs of the Italian ministry of health (response rate: 38%) National based cross-sectional study	To determine and evaluate professional activity (and the related skills and competencies) and the information demands and/or education and training needs of OPs; (self-administered questionnaire with 3 different sections; total of 35 questions: 1. personal and professional information; 2. training and updating need (scale variable from 1 = very unimportant to 5 = very important); 3. professional activity and practice characteristics)	(a) The Italian continuing medical education (CME) program is not considered to be sufficiently adequate to ensure effective updating of OPs; significant improvement could be achieved by training events discussing topics and issues that really met the practical needs of OPs or reducing the costs or the distance (< 100 km) of training events; higher training offer regarding the manual handling of loads (MHL), chemical substances, upper limb biomechanical overload, carcinogens and work-related stress; (b) need to achieve a better cooperation between general practitioners and OPs or other professions
Plomp and van der Beek [3] (2014) The Netherlands	n = 797 OPs (response rate: 45%) Cross-sectional study	Difference of desired and actual job perception and dissatisfaction factors in the settings: (1) occupational health service; (2) employed in the company; (3) self-employment (online questionnaire: 11 items per survey item with five-point Likert scales)	(a) Self-employed OPs show the highest job satisfaction on average; they are particularly satisfied with: financial compensation, personal responsibility and job security; (b) OPs of the occupational health service show on average the highest job dissatisfaction; they are particularly dissatisfied with the recognition of their work; (c) OPs employed in the company show medium satisfaction in almost all points (d) highest dissatisfaction of all groups concerns work pressure; (e) further dissatisfaction factor mentioned: poor image of the profession; (f) commercialization of the profession as a negative influence on being able to perform work in compliance with professional standards; (g) biographical variables (such as age or gender) without any influence on the job (dis-)satisfaction of OPs; (h) strongest influence on overall job satisfaction: level of autonomy and intrinsic/social aspects; (i) highest negative impact on job satisfaction: lack of professional challenges, high administrative burden and poor public image of the profession
Rodriguez-Jareno et al. [55] (2017) Spain	n = 168 OPs being members of the Catalan society of safety and occupational medicine (response rate: 57.9%-> representing 40.3% of the reference population) Cross-sectional study	To analyze the medical practice of workers’ health examinations in Catalonia (Spain) in terms of its occupational preventive aim (self-developed online survey: Likert-type scales with 4 or 5 categories, numeric text boxes for continuous variables and open boxes for comments)	(a) Health professionals from the external OHS dedicated more time, did 2.5 times more health examinations and had nearly 3 times more workers assigned to them; (b) less than half of participants had adequate and sufficient administrative support; (c) accessibility of workers to the external OHS was low, with 26% of employees making consultations outside health examinations for health problems possibly related to work, compared to 90% in internal services; (d) if additional tests/investigations specific to occupational hazards had to be requested, physicians in external services had significantly more difficulty obtaining them due to administrative/bureaucratic and/or commercial/financial reasons; (e) regarding awareness of sickness absence data, 6% of physicians from the external OHS had knowledge of work-related absences, and 3% had knowledge of non-work-related absences, compared to 75% and 49%, respectively, from internal services; (f) physicians made recommendations to the companies following health examinations but they were reportedly taken into account by companies in fewer than 2/3 of the cases
Verger et al. [56] (2010) France	n = 20 OPs (unknown response rate) Qualitative survey	Knowledge, attitudes and practices of OPs towards occupational cancers and perceived barriers to prevention (individual interviews using semi-structured interview guides, qualitative content analysis)	In general: (a) the majority of surveyed OPs see prevention of occupational cancers as part of their role (n = 15); (b) full-time OPs report less autonomy to act (n = 5); (c) a minority of OPs (n = 5) appear to prefer prevention that goes beyond the legal framework; (d) due to lack of time and resources: less time for occupational health activities per company than is actually required by law (n = 7); (e) low participation of workers in the prevention of occupational cancers (n = 15) for those working at the occupational health service: (a) lack of independence (n = 8); (b) little room for maneuver/ little scope for action (n = 10); (c) dependence on the employer; danger of own professional existence
Zaman et al. [57] (2017) The Netherlands	n = 13 OPs (unknown response rate) and n = 8 cancer patients (unknown response rate) Qualitative study with a cross-sectional descriptive design	To evaluate the feasibility of OPs trained in oncological work-related problems, and in providing work-related support to cancer patients within the curative setting (semi-structured interview with predefined topic list)	(a) The most frequently mentioned facilitator was ‘being more independent than an OP in the company’; (b) positive feedback from health care providers and patients about the received care and support that the OPs had given, and the additional knowledge of the OPs about cancer and work-related problems; (c) working within the clinical setting or outpatient clinic gives the opportunities to cooperate with other health care disciplines; (d) major barriers: lack of financial support for the OPs, unfamiliarity of patients and health care providers with the specialized OP; (e) OPs are not structurally embedded in the health care system; (f) non-optimal timing/scheduling of the consultations

**Table 4:** Identified work-related resources.

Top Category	Description/Definition	Attribution	N_Total—Assigned Studies
	Resources (Condition-Based)		
Social interaction	Processes of mutual exchange or reciprocal influence between different persons or social groups and the resulting appraisal.	Cooperation with other disciplines in the company	2
		frequent cooperation with other relevant stakeholders (healthcare staff, employers and social insurance agency)	2
		Accompanied consultations (e.g., spouse)	1
Reputation of the profession	The assessment of the occupational profile of “OP” by external parties and oneself.	Social recognition	1
		Professional/occupational acceptance	2
		Holding a specialty in occupational medicine	2
		Recognition of the meaningfulness of one’s own activity	1
Characteristics of the employment relationship	The possibilities of an employment relationship of OPs and associated characteristics.	Work experience/years of work	4
		High number of employees to be supervised	1
		Self-employed activity	3
		To be employed within the company	1
		Secondary occupation/part-time job	1
		Membership of profression-related research faculty	1
(Personnel) development and promotion	Incentives (on the part of the employer) that bind the OP to the work environment in perspective and maintain his or her willingness to be employed	Low competition	2
		Good development opportunities	1
		High job security	3
		Being more independent	1
		Reasonable, financial compensation	3
Organizational policy	Rules/regulations within the organizational procedures.	Well-established workplace policy regarding sickness certification	1
Scope for decision-making/action	According to the JDC model: a potential response to job demands that may be present in varying degrees in the workplace environment.	High degree of decision-making freedom	1
		High level of personal responsibility/ autonomy	1
Aspects of health	Factors that contribute in a direct way to complete physical, mental and social well-being as well as prevent the development of disease or infirmity.	Relatively positive health behavior	1
		Lower susceptibility to burnout	2
		Relatively low number of quantitative and emotional challenges	1
Work-life balance	Aspects that allow a successful work-life balance.	Successful balance between work and private life	2
		Average working hours	1
Total			42

**Table 5:** Identified work-related stress factors.

Top Category	Description/Definition	Attribution	N_Total—Assigned Studies
	Loading Factor/Stress Factors (Condition-Based)		
Social interaction	Processes of mutual exchange or reciprocal influence between different individuals or social groups and the resulting appraisal.	Cooperation among OPs	3
		Deficits in communication and cooperation with other professions, e.g., general practitioners	3
		Low social interaction at the workplace	2
Reputation of the profession	The assessment of the occupational profile of “OPs” by external parties.	Professional esteem/lack of acceptance by employers and employees	1
		Lack of social recognition	4
		Prejudices	1
Characteristics of the employment relationship	The possibilities of an employment relationship for OPs and associated characteristics (e.g., specific fields of work).	Occupation of OP > 41 years	1
		Employment in a small town or village	1
		Employment with occupational health service	4
		Main occupation	2
Socioeconomic factors	Economic, structural and social factors that significantly influence the work of OPs.	Insufficient budget expenditures for the occupational medicine department	2
		Uneven distribution of employed occupational physicians according to federal states	1
		Barriers for getting adequate training offers	1
		Unfamiliarity of patients and health care providers with specialized occupational physicians	1
		Structural barriers	1
		Commercialization of occupational medicine services	1
Perspectives	The view of future developments in the work of OPs.	Shortage of junior staff	2
		Decline in the number of employed occupational physicians	2
Information deficits	Characteristics that specifically indicate a lack of education or from an inadequate stream of information/communication.	Fear of consequences of a diagnosis of occupational disease	1
		Difficulty in diagnosing occupational diseases, somatoform and age-related diseases	3
		Inadequate studies/training in occupational medicine and occupational health	3
		Insufficient information, further training and education opportunities	5
		Difficulty regarding mangement of workplace safety and risk assessment	1
		Difficulty by prevention of health hazard due to overwork	1
		Difficulty of diagnosis of return to work	1
		Insufficient knowledge/experience of psychiatric expertise	2
Organizational complications	Potential conflicts and complications arising from the workplace environment and existing between the OP and the organization as well as the actors acting within it.	Subordination to the employer	2
		Resistance to change within the company	3
		Difficulty in measuring the success of implemented interventions	2
		Low involvement of employees in prevention tasks	1
		Lack of time and resources	3
		Utopian expectations on the part of employers	1
		lack of adequate and sufficient administrative support	1
		Work pressure	2
		Lack of professional challenges	1
Uncertainty factors	Undetermined factors that may place the acting OP in potential conflict situations and/ or conscience constraints.	Role conflict	1
		Ethical issues	1
		Difficulty diagnosing somatoform and age-related disorders	1
Professional obligations	General obligations which are associated with the occupational physician’s activity.	Handling sickness certifications	2
		High administrative burden	2
Scope for decision-making/action	Scope for decision/action according to the JDC model: A potential response to work demands that may be present in the workplace environment to varying degrees	Little room for maneuver/low autonomy of action	1
Total			74

## Data Availability

The datasets used and/or analyzed during the current study are available from the study group on reasonable request. Please contact the corresponding author.

## Abbreviations

ANHOPS	Association of National Health Occupational Physicians
BauA	Federal Institute for Occupational Safety and Health (dt.: Bundesanstalt für Arbeitsschutz und Arbeitsmedizin
CL	Consultation Letter(s)
CME	Continuing Medical Education
COPSOQ	Copenhagen Psychosocial Questionnaire
FFOM	Fellow of the Faculty of Occupational Medicine
FOM	Faculty of Occupational Medicine
GP	General Practitioner(s)
JBI	Joanna Briggs Institute
JDC	Job Demand Control
JSOH	Japan Society for Occupational Health
MD	Mean Deviation
MHL	Manual Handling of Loads
MFOM	Member of the Faculty of Occupational Medicine
MJSS	Minnesota Job Satisfaction Scale
MUPS	Medically Unexplained Physical Symptoms
NHS	National Health Service
OHS	Occupational Health Service
OP(s)	Occupational physician(s)
PCC	Population Concept Context
PRISMA P	Preferred Reporting Items for Systematic Review and Meta-Analysis Protocols
PRISMA ScR	PRISMA extension for Scoping Reviews
RCT	Randomized Controlled Trial
SF-36	Short Form 36
SME(s)	Small and Medium-sized Enterprise(s)
UK	United Kingdom

## Appendix A

**Table A1:** List of included studies and data extraction. Table A1 includes “Reference”, “Sample”, “Sociodemographic Data”, Research Design” and “Level Of Evidence”.

Reference, Country of Origin	Sample	Sociodemographic Data	Research Design	Level of Evidence
Alaguney et al. [36] (2020) Turkey	n = 478 physicians of which: n = 251 physicians who can demonstrate past or present work experience as an OP (response rate: 10.34%)	Average age: 49 years Employed in occupational health services: 64.8%	Cross-sectional study	IV
Cakir and Ilhan [37] (2018) Turkey	n = 258 active working OPs in Ankara (response rate: 100%)	Average age: 51.5 years Male: 83.7%; female: 16.3% Working in occupational health services: 68.2%; working fulltime: 10.9%; self-employed: 14%; working part-time: 7%	Cross-sectional study	IV
Demou et al. [38] (2018) International	Rating-round 1: n = 332 OPs (occupational physicians) (unknown response rate) Ranking-round 2: n = 232 OPs (unknown response rate)	Area of practice: (1) health care sector (physicians): 40.9% (2) industry (manager/physicians): 44.6% (3) academic (physician): 23.7% First round (rating): occupational groups/job title: (1) physicians: 68.7%; (2) manager/physicians: 18.1%; (3) academic/physicians: 11.7%; second round (ranking): occupational groups/job title: (1) physicians: 71.1%; (2) manager/physicians: 18.5%; (3) academic/physicians: 9.9%; both rounds (first + second): (1) average age (all occupational groups): 45–64 years; (2) male (all occupational groups): 66.7%; female (all occupational groups: 33.3% No statistically significant differences in the distributions of: gender, age, group, job practice and years of experience (for all: *p* > 0.05); between first and second round respondents within each occupational group	Delphi survey (second rounds over all) first round: rating second round: ranking (questionnaire-based) first and second round: same pool of persons, but irrespective of whether they had taken part in round 1 or not	IV
Glaser et al. [39] (2015) Austria main study	n = 147 OPs (unknown response rate)	Average age: 51.9 years Male: 35.4%; female: 64.6% Working in occupational health services: 35.4%; working fulltime: 48.3%; self-employed: 44.2%; working part-time: 51.7%	Cross-sectional study (online questionnaire)	IV
Glaser et al. [39] (2015) Austria pre-study	n = 6 OPs (unknown response rate)	Male: 50%; female: 50% Working in occupational health services: 33%; full-time employee in the company: 17%; working full-time: 83%; self-employed: 33%; working part-time: 27%; area of practice (science): 17%	Qualitative cross-sectional study	IV
Gross et al. [40] (2012) England	n = 145/224 NHS (national health service) OPs (England, Scotland, Wales) with responsibilities for health care workers; ANHOPS membership (response rate: 65%)	Average age: 49 years (SD = 9.1; range 28–76) male: 55%; female: 45% Average years of working for NHS: 9.6 years; OHPs provided services to an average of three different NHS trusts; combined average of 8804 employees	Cross-sectional study (postal questionnaire-based)	IV
Gyo et al. [41] (2016) Germany	n = 136 (1992) to n = 86 (2012) OPs in Germany (unknown response rate)	Not specified (ns)	Descriptive correlation study	III
Hobson et al. [42] (2016) England	n = 2 OPs working in the private sector OHS 108 accompanied consultations matched to 103 non-accompanied consultations (unknown response rate)	No further information on OPs; accompanied patients and control patients (both OPs) Average age: 46.5 years Male (both groups and OPs): 41%; female (both groups and OPs): 59% working in public sector: 74.5%	Prospective, unblinded, observational (over the course of 16 months)	III
Hoedeman et al. [43] (2010) The Netherlands	n = 43 OPs (response rate: 97.2%) as well as n = 489 sick workers with mild and severe medically unexplained physical symptoms (MUPS) (unknown response rate)	Average age: 46.5 years Male: 45.2%; female: 54.8% Working in occupational health services: 100%	Cross-sectional study	IV
Hoedeman et al. [44] (2010) The Netherlands	n = 6 RCTs (randomized controlled trials) with a total of 449 patients; RCTs concerning CLs for patients with MUPS (medically unexplained physical symptoms) (unknown response rate)	Not specified (ns)	Qualitative study (intervention/systematic review)	IV
Koike et al. [45] (2019) Japan	OPs working in full-time: 2002–2004: n = 578 to 2012–2014: n = 953 (unknown response rate)	Not specified (ns)	Cohort study/ longitudinal study	III
Lalloo et al. [46] (2020) Scotland	n = 213/1207 practicing UK OPs (response rate: 18%)	Some OPs worked across more than one practice area (variables are in accord with results)	Online survey	III
Lesage et al. [47] (2013) France	n = 1670/5010 OPs working in France (by French ministry of labor) (response rate: 33%)	Average age: 52.6 years Male: 29%; female: 71% Working for an independent health service provider: 77%; working for an in-house service: 23%	Nationwide cross-sectional study (online questionnaire)	III
Ljungquist et al. [48] (2015) Sweden	n = 481 OPs; n = 4257 GPs (general practitioners); n = 9452 physicians working in other clinical settings; overall: n = 22,349 physicians (not all included in the 3 groups above) (response rate: 60.6%); questions mailed to all of the 36,898 physicians working and living in Sweden (October 2008) all physicians who stated they had consultations concerning sickness certification at least once a month constituted the study group (n = 14,190)	OPs: age (20–44 years): 4.4%; (45–65 years): 84.6%; (>65 years): 11% Male: 59.7%; female: 40.3% Specialist: 95.2%; non-specialist: 4.8% Number of years at current workplace: (<5 years): 48.9%; (5–9 years): 31.1%; (>= 10 years): 19.9% Frequency of sickness certification consultation: (> 20 times a week): 14.8%; (6–20 times a week): 60.5%; (1–5 times a week): 20.1%; (about once a month: 3.7%	Cross-sectional study (paper-based)	IV
Moriguchi et al. [49] (2013) Japan	n = 557 OPs; Kyoto occupational health promotion center; (response rate: 31% (175 OPs); n = 76 no longer active as OPs; n = 86 OPs who were either; private clinic-based or hospital-based	Age (in 30’s): 3%; (in 40’s): 5%; (in 50’s): 26%; (in 60’s and over): 56% Male: 77 (90%); female: 9 (10%) Private practitioners: 64; physicians in hospitals: 22 Length of clinical practice experience: M = 33.4 (SD = 9.7 years) Experiences as OPs: M = 13.4 (SD = 8.3 years); served in plants: M = 6.2 (SD = 8.1 h/month)	Questionnaires via mail in 2008	
Moriguchi et al. [50] (2020) Japan	n = 181 OPs; 2016: n = 946 OPs/postal addresses; overall: n = 363 responses; (response rate: 38%); of these, n = 139 no longer active as OPs; other cases excluded; usable answers: n = 181 OPs: (50% of the 363 respondents or 19% of the original 946 mail addresses were usable); work setting: private clinic-based: 131 OPs; hospital-based: 50 Ops	Age (in 30’s): 3%; (in 40’s): 10%; (in 50’s): 31%; (in 60’s and over): 57% male: 153 (87%); female: 23 (13%) Not made clear: 5 cases; private practitioners: 131; physicians in hospitals: 50 Length of clinical practice experience: M = 32.3, SD = 10.8 years (median = 32 years); most of respondents were specialized in general practice or internal medicine; all of OPs in 2016 were certified OPs; 6 had certification of the occupational health consultant; no OPs with JSOH certification; no difference between active OPs in 2008 and 2016 in demographic and other patterns	Questionnaires via mail in 2016 (and similar survey in 2008)	IV ?
Moßhammer et al. [51] (2012) Germany	n = 23 primary care physicians/OPs; (unknown response rate)	Average age: 54 years Male: 75%; female: 25% Working in occupational health services: 25%; working full-time in the company: 37.5%; self-employed: 25%; working part-time: 12.5%	Qualitative cross-sectional study	IV
Nübling et al. [52] (2007) Germany	n = 356 OPs; (unknown response rate)	Not specified (ns)	Cross-sectional study with the addition of comparative data from different occupational groups	II–III
Nübling et al. [53] (2014) Germany	n = 777 OPs; (response rate: 34%)	Average age: 54 years Male: 49%; female: 51% Working in occupational health services: 40%; working full-time: >90%; self-employed: ca. 33%; working part-time: <10%	Cross-sectional study adding the reference groups “physicians in hospitals” and “average of all professions in Germany”	II–III
Persechino et al. [54] (2016) Italy	Random sample of 1237 OPs, enrolled in the national register of OPs of the Italian ministry of health; (response rate: 38%)	Age (<35 years): 2.7%; (35–44 years) 22.8%; (45–54 years): 23.6%; (55–64 years): 40.6%; (≥65 years): 10.3% Male: 72.4%; female: 27.6% Company size (served as OPs): <10 workers: 31.5%; 10–49 workers: 39.2%; 50–249 workers: 14.9%; ≥250 workers: 14.4% A third of respondents work exclusively as OPs	National based cross-sectional study	IV
Plomp and van der Beek [3] (2014) The Netherlands	n = 797 OPs; (response rate: 45%)	Average age: 51 years Male: 65.2%; female: 34.8% Working in occupational health services: 66.3%; full-time employed in the company: 9%; self-employed: 17.2%	Cross-sectional study	IV
Rodriguez-Jareno et al. [55] (2017) Spain	n = 168 OPs who were members of the Catalan society of safety and occupational medicine (response rate: 57.9%-> representing 40.3% of the reference population)	Average age: 47.3 years Male: 40.5%; female: 59.5% Working in the external OHS: 47.6% (n = 80); working in internal services: 52.4% (n = 88) Occupational physicians worked an average 36.8 h/week (median: 38), and spent between 64% (internal) and 84% (external) of their working hours in activities related to health surveillance	Cross-sectional study	
Verger et al. [56] (2010) France	n = 20 OPs; (unknown response rate)	Male: 30%; female: 70% Working in occupational health services: 40%; full-time employed in the company: 60%	Qualitative survey	IV
Zaman et al. [57] (2017) The Netherlands	n = 13 OPs (unknown response rate) and n = 8 cancer patients (unknown response rate)	Mean age of OOPs: 55 years Male: 54%; female: 46% Mean of years working as a regular OP:: 18	Qualitative study with a cross-sectional descriptive design	IV

**Table A2:** List of included studies and data extraction. Table A2 includes “Reference”, “Outcome (Objectives/Aim)”, “Results”, “Indentified Loading Factors/Stress Factors, “Identified Resources” and “Strengths and Weaknesses of Study”.

Reference, Country of Origin	Outcome (Objectives/Aim)	Results	Identified Loading Factors/Stress Factors	Identified Resources	Strengths and Weaknesses of Study
Alaguney et al. [36] (2020) Turkey	Underreporting of occupational diseases (online questionnaire: 30 questions and statements with five-point Likert scales from 1 = not important to 5 = very important)	(a) Fear of potential job loss as a result of occupational disease screening and diagnosis; for themselves (*p* = 0.015); for workers diagnosed with an occupational disease (*p* = 0.015); (b) inadequate examination of occupational safety and health and limitation of detection opportunities of occupational diseases in the workplace environment (*p* = <0.001); (c) insufficient awareness among physicians of work-related diseases and their under-reporting (*p* = 0.043)	(1) Fear about consequences after diagnosis of an occupational disease; (2) limited scope for action and expertise regarding work-related diseases	Work experience/years of work	Weaknesses: not mentioned by the authors
Cakir and Ilhan [37] (2018) Turkey	Working conditions (questionnaire: 85 questions), of which: Intrinsic, extrinsic and general job satisfaction (20 questions: Minnesota job satisfaction scale (MJSS) consisting of five-point Likert scales ranging from 1 = very dissatisfied to 5 = very satisfied)	(a) Average monthly working time of an OP: 143 h (36 h/week); (b) 80.6% of OPs consider their work to be suitable and meaningful; (c) 27.1% rate their occupational physician education/training as satisfactory or sufficient; (d) 86.3% of the respondents perceive the subordination to the employer as unpleasant; conflict of interest between payment and self-determined action; restriction of freedom of decision; (e) M (Intrinsic Satisfaction) = 3.5 M (Extrinsic Satisfaction) = 3.3; (f) significant positive relationship between extrinsic satisfaction and monthly salary (*p* < 0.001); (g) significant positive relationship between number of employees to be under an OP’s care and overall satisfaction (*p* = 0.013; (h) significant positive relationship between years worked as OP and intrinsic satisfaction (*p* = 0.009)	(1) Inadequate occupational medical or occupational health education/training; (2) subordination to the employer	(1) Work experience/years of work; (2) reasonable, financial compensation; (3) average working hours; (4) number of employees to be under a OP’s care; (5) recognition of the meaningfulness of one’s own profession	
Demou et al. [38] (2018) International	Job requirements and practice competencies by activity area, systematically developed questionnaires. Rating: ranking the importance of 12 competency areas for the individual activity (1 = not important to 5 = very important) Ranking: generation of a ranking of the previously listed areas with the addition of newly proposed items from round 1)	First round (rating): (a) most important competence for all groups: good clinical care (M = 4.56, SD = 0.20); (b) least important competence for all groups: teaching and educational supervision (M = 3.81, SD = 0.09) Second round (ranking): (a) top 3 rankings of all groups: (1) good clinical care; (2) general principles of assessment and management of occupational hazards to health; (3) assessment of disability and fitness for work; (b) lowest rankings of all groups: (1) management competences; (2) competencies on teaching/educational supervision (teaching and educational supervision); (c) for all other areas, there are clear differences according to the individual areas of occupational activity; the academic/physician group deviates most clearly from the other two groups; (d) consent: all 12 domains were regarded as important (90% and over)	inadequate occupational medical or occupational health education/training by scope of activities		Weaknesses: relatively low response rate; stronger European response (bias?); self-reported job titles (and high degree of crossover in OH practice)
Glaser et al. [39] (2015) Austria main study	Everyday work; work tasks; general loads/stress factors; cooperation with other occupational groups; professional identity; (online questionnaire with 221 items consisting of five-point Likert scales from 1 = no, not at all to 5 = yes, exactly)	everyday work/work tasks/general loads/stress factors: (a) dissatisfied with the range of training/education opportunities (n = 128); (b) lack of young professionals (M > 3); low status/relevance of occupational medicine in medical study/training (M = 4.2); (c) resistance to change on the part of employers (M > 3.5); (d) difficulty in measuring the success of implemented interventions (M > 3.5); (e) high documentation effort (M > 3); (f) competitive situation in occupational health services higher (M > 3) than for self-employed (M < 3; competition overall (just) little stressful; (g) different burdens according to employment relationship cooperation with other occupational groups: (a) classification/rating of cooperation as “meaningful” (M > 4); (b) competition with safety professionals and occupational psychologists is low (M < 2) professional identity: (a) rather dissatisfied with appreciation on the part of society (M < 3); (b) relatively satisfied with appreciation from employees (M > 3)	(1) Insufficient information and training opportunities; (2) resistance to change within the company; (3) difficulty in measuring the success of implemented interventions; (4) lack of junior staff; (5) high administrative burden; (6) type of employment: working for the occupational health service; (7) lack of social recognition	(1) Cooperation with other disciplines; (2) low competition; (3) professional appreciation; (4) work experience/years of work; (5) type of employment: self-employment	
Glaser et al. [39] (2015) Austria pre-study	Everyday work; work tasks; general loads/stress factors; cooperation with other occupational groups; professional identity (expert interviews using a deductively created interview guide; qualitative content analysis)	Everyday work/work tasks/general loads/stress factors (mentions): (a) insufficient information which is necessary for work (n = 3); (b) role conflict between reporting and confidentiality (n = 2); (c) payment in relation to workload is adequate (n = 4); (d) insufficient acceptance by employers and employees (n = 2); (e) initiation and implementation of changes in the company is problematic (difficulty in measurement of success of health promotion measures in the company or possible effects only measurable very late) (n = 2); (f) lack of junior staff (n = 4); (g) other named loads/stresses: physical stress, weather aspects, no clearly defined role as OP and difficulties in communicating/teaching about prevention in relation to the workplace Cooperation with other professional groups (mentions): (a) positive evaluation of cooperation (n = 3); (b) cooperation among OPs rather negative (n = 3) Professional identity (mentions): (a) competencies to bring along: impartiality, expertise and openness (n = 5); (b) utopian expectations on the part of employers (n = 5); (c) professional appreciation (n = 2); (d) social recognition (n = 4)	(1) Insufficient information; (2) role conflicts; (3) resistance to change within the company; (4) difficulty in measuring the success of implemented interventions; (5) lack of junior staff; (6) cooperation among OPs; (7) professional appreciation/lack of acceptance on the part of employer/employee; (8) utopian expectations on the part of employers	(1) Appropriate financial compensation; (2) cooperation with other disciplines; (3) social recognition	
Gross et al. [40] (2012) England	Determine the experience and training in identifying substance misuse among health care workers	(a) Only a small proportion of OHPs felt adequately trained in the assessment (39%), detection (37%) or treatment (12%) of substance misuse, and few used standardized addiction screening tools or brief interventions in routine practice; (b) occupational health physicians who participated in this survey were unfamiliar with dedicated services for addicted health care professionals and with local specialist NHS addiction services, and felt resources and support available to them were limited	Limited support (insufficient training and inadequate support) regarding substance-use problems of health care workers		Weaknesses: only a snapshot of training and experience of ANHOPS members from 2006; adequate representation of all doctors working in NHS OH departments; self-report nature of questionnaire
Gyo et al. [41] (2016) Germany	Number of state-certified OPs in relation to socio-economic data in Germany (manual research on various internet platforms or databases)	(a) Sharp decline in the number of OPs in Germany from 1992 to 2012 (decline rate: 37%); (b) positive correlation between the decrease in OPs and the increase in GDP in Germany (r = 0.47); government expenses concentrate on other areas; (c) uneven distribution of employed OPs in the discrete federal states (2012): Saarland: 8 OPs per 1 million employees; North Rhine-Westphalia: 0.8 OPs per 1 million employees	(1) Decline in employed OPs; (2) inadequate budgetary expenditures for the occupational medicine department; (3) uneven distribution of employed OPs by federal state		
Hobson et al. [42] (2016) England	A total of 108 accompanied consultations matched to 103 non-accompanied consultations; ill health retirement; diagnosis; complexity; time (duration); consultation process indicators (interruption, additional information, recording); the consultations occurred in clinics held in a number of different locations and included referrals from the public and private sectors and from a variety of workplaces. Public sector referrals were predominantly but not exclusively from two large local authorities	(a) Accompanied consultations more likely to be connected with: ill health retirement (*p* < 0.01); neurological diagnosis or multiple diagnosis (*p* < 0.01); rated as complex (*p* < 0.01); taking longer than 30 min (*p* < 0.01); (b) 54% of companions were spouse/partner (of patient); (c) an impact by the companion was recorded in 81% of consultations (but most frequently they had provided information (56%); (d) in 36% of consultations the impact of the companion was helpful or in agreement with the advice provided by the OPs; (e) in 28% of accompanied consultations interruptions were recorded; (f) 6% of consultation: consultation or companion was difficult; (g) 10 accompanied consultations where companion was a trade union representative: male: 80% (*p* < 0.05); but only 12 % of consultations were rated as complex; half of the consultations: trade union representative provides information; in 30% of consultations: interruptions and asked questions		Accompanied consultations (54% spouse or partner of patients) to deliver better understanding through more information	Weaknesses: selection bias: only two OPs; non-experimental
Hoedeman et al. [43] (2010) The Netherlands	Consultation load; difficulties and needs of OPs in the course of sickness certificate of employees with severe MUPS (Utrecht burnout scale and Utrecht work engagement scale)	(a) OPs do not need more time for workers with severe MUPS than for workers with low MUPS (*p* = 0.266); (b) communication difficulties with the treating primary care physician for workers with severe MUPS (OR = 5.42, *p* < 0.01); (c) relevant confounding factors: attribution of physical symptoms to somatoform causes (*p* = 0.005) and to patient age (*p* = 0.029)	(1) Deficits in communication and cooperation with the treating primary care physician; (2) insufficient knowledge of psychiatric expertise/knowledge; (3) difficulty in diagnosing somatoform disorders; (4) difficulty in diagnosing age-related diseases		Strengths: diversity (urban/rural population in different branches and differently sized organizations); validated questionnaires (gathered from OPs and employees by means) Weaknesses: no conclusions regarding causal relationships; self-report questionnaires; no additional medical check on the MUPS; no question about whether OPs diagnosed somatization, depression, and anxiety as MUPS; no qualitative analysis of Ops’ answers with regard to diagnosis, task difficulties and their own characteristics
Hoedeman et al. [44] (2010) The Netherlands	A total of 2 authors screened the abstracts of the studies + independently assessed: the risk of bias of the included studies Primary outcome measures: health care (provider)-related; patient-related secondary outcomes (patient-related): sick leave and return to work; functional status (SF-36); depression and anxiety (e.g., interview, Beck depression inventory) Objectives to assess the effectiveness of consultation letters (CLs) to assist primary care physicians or occupational health physicians (OPs) in the treatment of patients with MUPS and diagnostic subgroups	None of the studies were performed in an occupational health setting and there were no data on sub-populations of employees, so no conclusions can be drawn on the effect of the intervention for employees regarding return to work or functioning at work; the results show an effect on improving physical functioning and a small effect on reducing social function, which can be of importance in the functioning and return to work of employees, but no conclusions can be drawn with regard to the exact effects Analysis of Consultation Letters (CL): (a) n = 4 studies (267 patients), intervention (CL following a consultation between patient and psychiatrist) resulted in: reduced medical costs (2 studies pooled for outcome (MD = −352.55 US$); improved physical functioning (3 studies pooled for outcome (MD = 5.71); (b) n = 2 (82 patients), intervention (CL following a joint consultation between patient and psychiatrist and physician) resulted in: reduced severity of somatization symptoms, reduced medical consumption, improved social functioning; (c) serious limitations in generalizability of the results to modern health care: most trials reported doctor-related outcomes with patient-related outcomes varying in results; the intervention appears to be far more effective for the most serious but rare disorders, and less so in the more common forms of MUPS; five of the six studies were performed in the United States and four studies before 1995; the studied populations were small and five of the six studies were of moderate quality Conclusion (authors): very limited evidence that a joint consultation with the patient by a psychiatrist in the presence of the physician, together with the provision of a CL, reduces severity of somatization symptoms and medical consumption; final conclusion: CL may be helpful for physicians who treat patients with MUPS (based on the provider-related outcomes). However, until further studies are conducted to find out if the intervention results in improved patient-related outcomes, the overall effectiveness of CLs cannot be demonstrated			
Koike et al. [45] (2019) Japan	Retention rate/trends of OPs and factors associated with it (semiannual survey dates through censuses of physicians from 2002 to 2014)	(a) Retention rate from 2012 to 2014: 76% (24% of OPs stopped working full-time); (b) the chance to continue working as an OP decreases when working in a small town or village (*p* < 0.05); (c) the chance to continue working as a OP decreases if the OP has been working for more than 41 years (*p* < 0.05); (d) the chance to continue working as an OP increases if an OP has already been registered as an OP in > 2 consecutive survey periods (*p* < 0.001)	(1) Decrease in the number of employed OPs; retention rate not saturated; (2) employment in a small town or village; (3) occupational physician employment: 41 years	Work experience/years of work	Strengths: large sample cohort; self-reporting (area of practice; no data for part-time OPs
Lalloo et al. [46] (2020) Scotland	Current and former research-activity; current and former teaching activity; demographics; qualifications; career profile; research related attitudes; FOM dissertation experience	(a) 162 (76%) undertook research at some career-point, of which 44 (27%) were currently research-active; (b) 154 (72%) undertook teaching at some career-point, of which 99 (64%) were currently teaching active; (c) of those who had never undertaken research (n = 51) or teaching (n = 59), 40 and 42% were interested in doing so; (d) key barriers: lack of time and opportunity; research activity was higher in healthcare OPs compared to industry OPs	(1) Lack of time higher in industry than healthcare OPs; (2) lack of statistical and supervisor support for dissertations, research experience and ethics application (lack of training and mentorship)	Member of the faculty of occupational medicine (MFOM) and fellow of the faculty of occupational medicine (FFOM) were more research-active	Weaknesses: potential biases by piloting and expert panel use in questionnaire development (Delphi study)
Lesage et al. [47] (2013) France	Maslach burnout inventory (emotional exhaustion, depersonalization, feelings of low personal accomplishment); perceived stress scale (stress level); primary appraisal of identity scale (identity threat; job characteristics	(a) 11.8% burnout compared to 5% in French general practitioners (main characteristic of the burnout pattern: feelings of very low personal accomplishment: 63.9%); (b) weak correlations with job characteristics; (c) stress and identity threat correlating with all three dimensions of burnout; (d) perceived stress-> main risk factor for emotional exhaustion and identity threat for feelings of low personal accomplishment	(1) increased numbers of workers to follow prevent OPs from performing all their tasks properly (feeling of unfinished work); (2) estimated prevalence of burnout and high rate of people at high risk of low personal accomplishment (higher than in most of the studies that have investigated other specialist groups)		Weaknesses: underrepresentation of OPs aged over 60 years; low response rates
Ljungquist et al. [48] (2015) Sweden	Work situation of occupational health physicians (OPs) regarding handling of sickness certification compared with other physicians, in particular general practitioners (GPs); associations between OPs’ experiences of assessing and providing a long-term prognosis of patients’ work capacity and some potentially interrelated factors; 163 questions about physician’s work with sickness certification mailed to home address; 11 items on sickness certification and general work	(a) Among OPs, a rather high proportion (46%) had a well-established workplace policy and substantial support from their immediate manager regarding sickness certification tasks, compared with GPs (32%) and especially with physicians working in other clinical settings (14%); (b) collaborations with other team members, with the Social Insurance Agency, and, most of all, with employers, was much more frequent among OPs than among GPs and among the other physicians (employers: 76%); (c) 43% of OPs found it problematic to handle sickness certification at least once a week (GP: 54%); (d) participation in coordination meetings with the SIO and/or employers concerning specific patients on a weekly basis was negatively associated with finding it ‘not at all/somewhat problematic’ to provide a long-term prognosis about patients’ work capacity; (e) OPs seem to have a more favorable work situation in their work with sickness certification; (f) experience of sickness certification consultations as problematic once a month or less often, not experiencing sickness certification tasks as a work environment problem, holding a specialty in occupational medicine, and having a well-established workplace policy regarding sickness certification matters were significantly positively associated with finding assessment of work capacity as ‘not at all/somewhat problematic’; (g) participation at least once a week in coordination meetings with the Social Insurance Agency and/or employer regarding sickness certified patients was negatively associated with finding assessing patients’ work capacity as ‘not at all/somewhat problematic’	Participating at least once a week in coordination; meetings with the Social Insurance Agency and/or employer regarding sickness certified patients	(1) Not experiencing sickness Certification tasks as a work environment problem; (2) having a well-established workplace policy regarding sickness certification matters; (3) OPs reported frequent cooperation with other healthcare staff, employers, and the Social Insurance Agency; (4) holding a specialty in occupational medicine	Strengths: all physicians in a whole country; relatively high response rate (61%) and large study group; questionnaire can be considered to have good validity Weaknesses: inability to detect the impact direction of associations found; not knowing how the questions were interpreted by the participants; not knowing how the non-responders would have answered the studied items; alternative models and explanations to our findings cannot be ruled out
Moriguchi et al. [49] (2013) Japan	Examine activities of private clinical- or hospital-based OPs; identify difficulties encountered in occupational health service	(a) OPs wished to allocate more time for: examination follow-up (2.6 h/month); mental health care (2.0 h/month); prevention of overwork (1.9 h/month); attendance at the safety and health committee meetings in the plant (1.9 h/month); (b) discrepancy between the current and the desired allocation was greatest for: risk assessment (171% as the desired/current ratio); maintenance and management of work and the work environment (150 and 152%); time allocation for health examinations appeared to be sufficient; (c) difficulties were experienced most often in: management of mental ill health (36 OPs); guidance of workers on sick leaves (11 OPs); followed by prevention of health hazard due to overwork (30 OPs); diagnosis of return to work (15 OPs); (d) many OPs had difficulty in dealing with: industrial hygiene-related issues such as risk assessment (14 OPs) and maintenance and management of work and work environment (11 cases each; (e) respondents were generally self-confident regarding: physical health management (typically providing general health examinations) except for a few specific health examination issues; to solve the problems related to lack of experience with mental health issues, proposals were made such as providing opportunity for exchange of information on these issues with experts for common sharing of experiences and for construction of a network	(1) Difficulty in dealing with industrial hygiene-related issues such as risk assessment, and maintenance, and management of work and work environment; (2) difficulties were experienced most often in management of mental ill health and guidance of workers on sick leaves; (3) followed by prevention of health hazard due to overwork; (4) diagnosis of return to work	(1) Generally self-confident regarding physical health management (typically providing general health examinations); (2) time allocation for health examinations appeared to be sufficient	Weaknesses: 12.5% of the enterprises served by the present 86 OPs had 300 workers and less, whereas the enterprises with more than 300 workers accounted for 8.3%; although a majority of the enterprises studied were of small-scale, the enterprise size of the present survey was somewhat skewed toward to larger ones; present study is biased in the distribution of types of industries studied
Moriguchi et al. [50] (2020) Japan	To compare the activities and encountered difficulties of Japanese part-time OPs in 2008 and 2016 and to investigate the effects of the stress-check program	(a) 62% OPs frequently encountered difficulties in the stress-check-related activities in 2016; (b) many OPs reported difficulties in the mental health care and the prevention of health hazard due to overwork both in 2008 and 2016; (c) enforcement of the stress-check program in 2015 changed the activities of part-time OPs in Japan; (d) OPs wished to allocate more time for: prevention of overwork (2.3 h/month); General health examination (2.1 h/month); stress-check (2.1 h/month); Follow-up of examination (1.9 h/month); round of the work area (1.8 h/month); interviews with high-stress employees (1.2 h/month); management of stress-check system (1.4 h/month); (e) discrepancy between the current and the desired allocation greatest for: development of comfortable workplaces (332% as the desired/current ratio); health promotion activity and health and hygiene education (271 and 267%); time allocation for fields related to periodical general health examinations appeared to be sufficient; (f) in 2016, difficulties were experienced most often in: stress-check (112 OPs); followed by mental health care (66 OPs); prevention of health hazard due to overwork (61 OPs); diagnosis of return to work (38 OPs); OPs encountered difficulties more in interview with high-stress employees than management of stress-check system; (g) proposals were made by 39 OPs: increase of training course for information exchange of experiences with experts (9 OPs); sharing roles of mental health issues with psychiatrists (8 OPs); 45 (25%) answered that training for JMA qualification was sufficient	(1) In 2016: difficulty in SC-related issues, especially in interview with high-stress employees; (2) not enough time in 2008 for plan and advice for occupational safety and health (OSH) policy; (3) attendance at the meeting of HS committee; (4) rounds of the work area; (5) risk assessment; (6) maintenance and management of work; (7) maintenance and management of work environment; (8) mental health care; (9) 2016 for preventive services reduced by enforcement of SC since 2015; (10) many OPs wished to increase the training course for information exchange of experiences on mental health issue with psychiatrists or highly professional OPs		Weaknesses: 12.1% of the enterprises served by OPs had less than 50 employees, enterprises with 50 employees and more accounted for 97.0% (although a majority of the enterprises studied were of small- and medium-scale, the enterprise size of the present survey was skewed toward to larger ones); although most of enterprises in Kyoto were small-size, situation of SC-related activities in those enterprises without OPs could not be investigated sufficiently in the present study; biased in the distribution of types of industries studied; while both musculoskeletal disorder and mental ill health were some of main symptoms reported by employees in Europe, musculoskeletal disorder issues were not specifically addressed in the present analyses; although return of employees to workplaces was discussed in general, specific needs such as post-stroke care were not addressed
Moßhammer et al. [51] (2012) Germany	Cooperation/communication among primary care physicians and OPs in Germany: deficits and barriers (focus group interviews using semi-standardized interview guidelines, qualitative content analysis)	(a) Existing deficits on the topics: work disability, chronic diseases and reintegration of workers; (b) mentioned/named barriers: fear, mistrust, prejudices, lack of legal regulations and lack of knowledge regarding the respective other occupational group; (c) view of OPs on prejudices and ways of dealing, e.g., “occupational physicians in the past, were those, either the women part-time or hourly, or the established ones had the impression: ‘he barely managed his studies, now he’s going to be an occupational physician.’ that was the view and [. . .], that’s how you were treated [. . .].”	(1) Deficits in communication and cooperation with primary care physicians; (2) existing barriers to successful communication and cooperation; (3) afflicted with numerous prejudices; corresponding clearance		Strengths: initial study presenting systematic gathered data regarding deficits in cooperation and barriers of primary care physicians and OPs; heterogeneity of composition and different groups Weaknesses: participants mainly long-time professional experience; selection bias maybe because of not responding doctors which are more distressed
Nübling et al. [52] (2007) Germany	(1) Perception of workplace; (2) consequences of stress; (3) psychosocial workplace factors (Copenhagen psychosocial questionnaire (COPSOQ) with comparative data from different occupational groups)	(a) OPs perceive a significantly lower conflict between work and private life than many of the comparative occupational groups (scale mean = 42); (b) the scale of social relationships at work is rated below average by OPs (scale mean = 36); (c) job insecurity is very low among the group of OPs (scale mean = 23); (d) OPs feel significantly less affected by burnout in comparison with other occupational groups (scale mean = 37)	Low social interaction in the workplace/at work	(1) Successful balance between work and private life; (2) job security; (3) lower susceptibility to burnout	
Nübling et al. [53] (2014) Germany	(1) Work requirements/work situation; (2) health behavior; (3) support wishes for health prevention; (4) stresses and loads (COPSOQ + further items on health and and job-specific stresses)	(a) Work-life balance represented a significantly more positive influence among OPs than among hospital physicians (scale mean of 44 to 72); (b) OPs have on average lower quantitative and emotional demands than hospital physicians; (c) quantitative requirements vary depending on the employment relationship: full-time and salaried OPs have higher quantitative requirements than self-employed and part-time OPs; (d) competitive pressure very low (mean scale value = 25); (e) scope for decision-making and development opportunities are very high among the OPs surveyed compared to the average of the comparison groups (scale mean = 67 and 77); (f) the level of job insecurity among OPs is very low (11 points compared to 32 points for the average of all occupations); (g) low level of social relationships during work (scale mean = 32); (h) lack of social recognition (scale mean = 59); (i) the overall health behavior of OPs is better than that of the general population: they pay more attention to a healthy diet (64 points compared to 51 points), exercise more (77 points compared to 40 points), and show a lower tendency to be overweight (40 points compared to 53 points); (j) lower risk of developing burnout (35 points versus 47 points for hospital physicians)	(1) Type of employment: primary occupation (full-time?); (2) low social interaction in the workplace; (3) lack of social recognition	(1) Successful balance between work and private life; (2) type of employment: self-employment/independently and part-time work; (3) low competition; (4) high degree of decision-making freedom; (5) good development opportunities; (6) job security; (7) relatively positive health behavior; (8) lower susceptibility to burnout; (9) relatively low number of quantitative and emotional demands	
Persechino et al. [54] (2016) Italy	To determine and evaluate professional activity (and the related skills and competencies) and the information demands and/or education and training needs of OPs; (self-administered questionnaire with 3 different sections; total of 35 questions: 1. Personal and professional Information; 2. training and updating need (scale variable from 1 = very unimportant to 5 = very important); 3. professional activity and practice characteristics)	(a) The Italian continuing medical education (CME) program is not considered to be sufficiently adequate to ensure effective updating of OPs; significant improvement could be achieved by training events discussing topics and issues that really met the practical needs of OPs (4.56) or reducing the costs (4.42) or the distance (< 100 km) of training events (4.28) (scale mean); higher training offer regarding the manual handling of loads (MHL) (15.1%), chemical substances (13.6%), upper limb biomechanical overload (12.2%), carcinogens (11.8%) and work-related stress (9.0%); (b) need to achieve a better cooperation between general practitioners and OPs or other professions	(1) High information demands, training and updating because of changes in workplaces and production processes (emergence of new occupational risks and diseases and modifications in regulatory framework for occupational health and safety); (2) barriers for the perception of training offers; (3) lack of cooperation with other professions		Weaknesses: only one survey time; no control/reference group; no causal conclusions possible; potential bias due to self-reporting; selection bias
Plomp and van der Beek [3] (2014) The Netherlands	Difference of desired and actual job perception and dissatisfaction factors in the settings: (1) Occupational health service; (2) employed in the company; (3) self-employment (online questionnaire: 11 items per survey item with five-point Likert scales)	(a) Self-employed OPs show the highest job satisfaction on average; they are particularly satisfied with: financial compensation, personal responsibility, and job security (0.7, 0.8, and 0.7 on the difference scale from −1: not satisfied to 1: very satisfied); (b) OPs of the occupational health service show on average the highest job dissatisfaction (e.g., intrinsic satisfaction: −0.6, autonomy: −0.2); they are particularly dissatisfied with the recognition of their work (−0.9); (c) OPs employed in the company show medium satisfaction in almost all points (e.g., intrinsic satisfaction: 0.0, autonomy: 0.2, personal responsibility: 0.0) (d) highest dissatisfaction of all groups concerns work pressure (M = 3.0–4.0); (e) further dissatisfaction factor mentioned: poor image of the profession (M = 2.6–3.9); (f) commercialization of the profession as a negative influence on being able to perform work in compliance with professional standards; (g) biographical variables (such as age or gender) have no influence on the job satisfaction or dissatisfaction of OPs; (h) the strongest influence on overall job satisfaction is the level of autonomy and intrinsic/social aspects; (i) highest negative impact on job satisfaction by: lack of professional challenges, high administrative burden and poor public image of the profession	(1) Type of employment: working for the occupational health service; (2) work pressure; (3) lack of social recognition; (4) commercialization of the profession; (5) high administrative burden; (6) lack of professional challenges	(1) Type of employment: self-employment; (2) financial remuneration/salary job security personal responsibility/ autonomy	
Rodriguez-Jareno et al. [55] (2017) Spain	To analyze the medical practice of workers’ health examinations in Catalonia (Spain) in terms of its occupational preventive aim (self-developed online survey: Likert-type scales with 4 or 5 categories, numeric text boxes for continuous variables and open boxes for comments were used for the answers)	(a) Health professionals from the external OHS dedicated more time, did 2.5 times more health examinations and had nearly 3 times more workers assigned to them (3709 workers/full-time physician vs. 1353 for those in internal services); (b) less than half of participants had adequate and sufficient administrative support; (c) accessibility of workers to the external OHS was low, with 26% of employees making consultations outside health examinations for health problems possibly related to work, compared to 90% in internal services; (d) if additional tests/investigations specific to occupational hazards (laboratory tests or others), not routinely included in the usual health examinations, had to be requested, physicians in external services had significantly more difficulty obtaining them due to administrative/bureaucratic and/or commercial/financial reasons; (e) regarding awareness of sickness absence data, 6% of physicians from the external OHS had knowledge of work-related absences, and 3% had knowledge of non-work-related absences, compared to 75% and 49%, respectively, from internal services; (f) physicians made recommendations to the companies following health examinations but they were reportedly taken into account by companies in fewer than 2/3 of the cases	(1) External service; (2) lack of adequate and sufficient administrative support; (3) companies do not accept recommendations of OHPs	Internal service	Weaknesses: only one survey time; no control/reference group; no causal conclusions possible: potential bias due to self-reporting; selection bias: physicians who did not participate in this study may have been different from that of the respondents weaknesses: no comparison was possible between participating and non-participating physicians who performed health examinations in their usual practice
Verger et al. [56] (2010) France	Knowledge, attitudes and practices of occupational physicians towards occupational cancers and perceived barriers to prevention (individual interviews using semi-structured interview guides, qualitative content analysis)	In general: (a) the majority of surveyed occupational physicians see prevention of occupational cancers as part of their role (n = 15); (b) full-time occupational physicians report less autonomy to act (n = 5); (c) a minority of OPs (n = 5) appear to prefer prevention that goes beyond the legal framework; (d) due to lack of time and resources: less time for occupational health activities per company than is actually required by law (n = 7); (e) low participation of workers in the prevention of occupational cancers (n = 15) for those working at the occupational health service: (a) lack of independence (n = 8); (b) little room for maneuver/ little scope for action (n = 10); (c) dependence on the employer; danger of own existence (professional)	(1) Type of employment: primary occupation (full-time) and working for the occupational health service; (2) lack of time and resources; (3) subordination to the employer; (4) ethical issues; (5) low involvement of employees in prevention tasks; (6) little room for maneuver/scope for action, low autonomy of action		strengths: sample intentionally diverse; social desirability bias; socio-cognitive bias (esp. in France); small sample size
Zaman et al. [57] (2017) The Netherlands	To evaluate the feasibility of OPs who is trained in oncological work-related problems, and in providing work-related support to cancer patients within the curative setting (semi-structured interview with predefined topic list)	(a) The most frequently mentioned facilitator was ‘being more independent than an occupational physician in the company’; (b) positive feedback from health care providers and patients about the received care and support that the OPs had given, and the additional knowledge of the OPs about cancer and work-related problems; (c) working within the clinical setting or outpatient clinic gives the opportunities to cooperate with other health care disciplines; (d) major barriers for being active as an OP were lack of financial support for the OPs and the unfamiliarity of patients and health care providers with the specialized occupational physician; (e) OPs is not structurally embedded in the health care system; (f) timing of the consultation is not yet optimal	(1) Lack of financial support; (2) unfamiliarity of patients and health care providers with the specialized occupational physician; (3) structural barriers	(1) Being more independent as OPs (than an occupational physician in the company); (2) positive feedback about care, support and additional knowledge; (3) working within the clinical setting or outpatient clinic (multidisciplinary cooperation)	Strengths: different survey groups; deductive guideline development; standardized evaluation procedure; second reviewer strengths/ weaknesses: qualitative expert opinions/self-assessment (no claim to representativeness)
